# Pandemic prevention should not victimize Indigenous Peoples and Local Communities

**DOI:** 10.1111/conl.12813

**Published:** 2021-05-31

**Authors:** Jason M. Tylianakis, Mark R. Herse, Sanna Malinen, Phil O'B. Lyver

**Affiliations:** ^1^ School of Biological Sciences University of Canterbury Christchurch New Zealand; ^2^ UC Business School University of Canterbury Christchurch New Zealand; ^3^ Manaaki Whenua Landcare Research Lincoln New Zealand

**Keywords:** bushmeat, COVID‐19, disease mitigation, equity, ethnic, Indigenous Peoples, pandemic, racial inequality

## 1 INTRODUCTION

During times of crisis, such as the present COVID‐19 pandemic, calls for immediate solutions are typically as rapid as the apportionment of blame. For example, the implication of wildlife consumption as a potential source of COVID‐19 (Cohen, 2020; Li et al., 2020) led China to ban the hunting, consumption, trade, and transport of wild animals (including those with important societal values) (Koh et al., 2021; Xiao et al., 2021). This reaction is similar to the bans that occurred in west Africa following the 2013–2016 Ebola outbreak (Bonwitt et al., 2018). Vietnam has issued a directive to strengthen existing penalties for illegal trade and consumption of wildlife. Moreover, hundreds of animal rights groups have signed an open letter to the World Health Organization calling for widespread bans internationally, framing wild meat as a luxury status symbol (Briggs, 2020). Total bans on wild animal trade and products fall at the extreme end of the policy continuum, with less‐extreme alternatives involving calls to specifically ban the trade of wild mammals and birds for consumption, or to close live animal markets (Roe et al., 2020). However, many restrictions of wildlife hunting, consumption, and trade disproportionately harm those in poverty and Indigenous Peoples and Local Communities (IPLC, defined by IPBES as ethnic groups who are descended from and identify with the original inhabitants of a given region, in contrast to groups that have settled, occupied or colonized the area more recently; IPBES 2019) (Booth et al., 2021; Roe et al., 2020). As has been noted previously, wild meat is the principal protein source, and animal trade is a key source of income, for millions of people (Booth et al., 2021; Cawthorn & Hoffman, 2015; Milner‐Gulland & Bennett, 2003; Roe et al., 2020), including many IPLC. So, without measures to balance the impact of such policy, many of the world's poorest people could suffer or die from highly restrictive harvest bans touted as “solutions.” Yet, in addition to the loss of food and income, and to socio‐economic conditions associated with high rates of COVID‐19‐related mortality (Ferrante & Fearnside, 2020), IPLC would be forced to carry a further cultural burden. Because harvest practices involving key species have underpinned many cultures for centuries, any policy mechanisms that inhibit or reduce harvest or consumption of wildlife have the potential to weaken culturally important connections of IPLC with the environment (Ellis et al., 2021; Lyver et al., 2019a). This disconnection can generate the perverse long‐term outcomes of disrupting cultural expression and transmission, and thereby further undermining both environmental and cultural resilience (Lyver et al., 2019a).

Here, we urge governments to reject the pressure to unilaterally ban wild meat and, if applying a more nuanced approach to wildlife harvest and trade restrictions, to ensure that policy recognizes the specific harm that can occur to IPLC. In particular, we argue that (1) there is inequity to prohibition of wildlife harvest and trade, both in terms of nutrition and income (Booth et al., 2021; Roe et al., 2020) and by carrying implicit trade‐offs among cultural imperatives. For example, we argue that (2) scapegoating wild meat harvests can divert attention from other key contributors to pandemic origin and spread, which may be more typically associated with wealthy and western consumption patterns or people as vectors. Finally, (3) if harvest or trade bans prove to be the optimal solution to pandemics like COVID‐19, these need to be balanced by investment into measures to protect income (e.g., increased land ownership rights, payment for ecosystem services, employment in wildlife management or nature‐based tourism; Cooney et al., 2017), prevent starvation (e.g., sustainable aquaculture, local‐scale farming of low‐disease‐risk species; Booth et al., 2021), and avert the disruption of culture. To address all these issues, pandemic prevention policy should be developed in partnership with IPLC.

Finally, recovery from crisis provides an opportunity to improve resilience to future crises, and we propose policies that reconnect people with their environments (rather than block connections through wild meat restrictions) to achieve this resilience. We outline the evidence for these arguments and build on recommendations from the Convention on Biological Diversity (2012), highlighting alternative policy directions for dealing with pandemic risk in a more equitable way.

## 2 THE INEQUITY OF HARVEST AND TRADE RESTRICTION

Whether at local or global scales, heterogeneity in the human population will determine the socioeconomic impact of any pandemic policy (Akbarpour et al., 2020). Clearly, overexploitation and wildlife trade risk disease transfer and threaten biodiversity. However, knee‐jerk policy can prioritize subsets of society and give primacy to dominant worldviews, thereby generating unexpected consequences for those whose voices were absent from its conception (van Vliet, 2018). For instance, millions of tonnes of bushmeat are consumed each year globally (Booth et al., 2021; Milner‐Gulland & Bennett, 2003), and wildlife trade provides crucial income and food security to many communities, such that many restrictions on this resource will disproportionately affect vulnerable groups (Roe et al., 2020). Among these communities, many IPLC have limited food sovereignty and security (Zavaleta‐Cortijo et al., 2020) and can become more dependent on wild meat when shocks like the COVID‐19 pandemic disrupt food supply chains and/or income sources (Lindsey et al., 2020).

In addition to its nutritional and economic value, harvesting of food is an important means through which many IPLC enact, maintain, and disseminate their culture (Berkes et al., 2000). Consequently, curbing the use of species threatens cultural wellbeing and connection to place, as well as local institutions and systems of ownership, rules and practices for management of species for food or medicinal use. Importantly, prohibition of hunting creates social and cultural feedbacks (in addition to the economic feedbacks discussed above) that harm IPLC and impact their resilience to future change (Lyver et al., 2019a). For example, bans on wildlife hunting would remove pathways for maintaining and transferring knowledge (e.g., hunting skills), community kinship, and social structures required to harvest food sustainably (Yletyinen et al., 2020). Moreover, the opportunity to experiment with alternative management responses following minor crises is crucial for making social‐ecological systems more resilient to larger catastrophes (Lyver et al., 2019a). Given that harvest is frequently a reason and mechanism for wildlife management by IPLC, restrictions on harvest will eliminate such experimentation, and thereby reduce the resilience of IPLC (and the ecosystems they manage) to further disruptions such as climate change. Moreover, such restrictions likely also violate rights protected by articles within the United Nations Declaration on the Rights of Indigenous Peoples (United Nations General Assembly, 2007). In extreme cases, enforcement of harvest bans has even been used internationally as a mechanism of cultural genocide (Crook et al., 2018). Even if trade, rather than harvest, bans are implemented, this can cause specific harm to IPLC. For example, trade of natural products contributes numerous societal functions such as reciprocity and respect between groups, transferring knowledge, reinforcing political agendas, and building alliances (Rout & Reid, 2019).

Finally, criminalizing IPLC and subsistence hunters who continue to hunt (Gombay, 2014) may force them to potentially subvert harvest activities into a “black market” (Challender & MacMillan, 2014). This would make tracking future wild animal trade (including for monitoring disease emergence and transmission) more difficult, and cause risks to conservation priorities (Roe et al., 2020; Roe & Lee, 2021). In addition to these public health and conservation risks, there is clear social harm caused by criminalizing culture (Carrington, 2011).

Based on these issues, we highlight several recommendations of the Convention on Biological Diversity (2012) that could be applied to protect against this specific harm to IPLC. Specifically, we advocate for policy that:

1. recognizes and reaffirms IPLC rights and responsibility for local wildlife and habitats, in which partnerships are established to empower IPLC institutions in the decisions about responses to mitigating disease spread;
2. enhances government and private sector support for IPLC to prioritize and protect the populations and habitats of bushmeat species that have lower zoonotic disease transfer risk, while alleviating the need for high‐risk species;
3. promotes a cultural‐precautionary policy approach that considers the deeper long‐term impacts of regulations on cultural integrity, such as identity, connection to place, language use, customary economies, customary practices, and knowledge systems;
4. supports IPLC to apply indigenous and local knowledge systems in policy and planning to find solutions that mitigate disease transfer risk;
5. grows capacity within IPLC to implement wildlife (and domestic) disease surveillance, sanitary control, and biosecurity measures to mitigate the spread of harmful pathogens.

## 3 WILD MEAT AS A SCAPEGOAT

Preventing future pandemics is obviously critical to minimize human suffering and economic loss. However, any time policy is enacted, there is potential for inequity and hypocrisy, and we worry that policymakers may not consider alternative solutions that hold the world's wealthy or dominant cultures accountable for their contributions to modern disease risk. For example, farmed meat is responsible for livestock‐originating disease transmission and pandemic risk to humans (Gray et al., 2007; Jones et al., 2013; Shortridge, 1992), as are meat processing plants (Middleton et al., 2020). However, despite this ongoing risk, farmed meat's typically wealthier consumers (Ritchie & Roser, 2017) are not subjected to its continued prohibition, as are those consumers of wild meat in China (even though temporary trade and consumption bans on farmed meat may occur following outbreaks of diseases such as foot and mouth).

In addition, deforestation and agricultural expansion increase human–wildlife encounter frequency and the risk of wildlife‐originating zoonotic disease transmission (both directly, and indirectly via wildlife–livestock encounters) along forest–farm boundaries, particularly in the tropics (Allen et al., 2017; Olivero et al., 2017; Wolfe et al., 2005). However, the economies and resource demand of high‐income countries, which disproportionately drive deforestation globally (Mills Busa, 2013), are not targeted by pandemic‐prevention policies. Curbing deforestation and urbanization of natural areas (Olivero et al., 2017; Wolfe et al., 2005) should therefore be an international policy priority for reduction of zoonotic disease risk.

Lastly, indiscriminate international travel by the wealthy facilitates the global spread of any pandemic, as seen during the current crisis, whereas policy focused on wild meat targets only one source of disease, not its global community transmission. It remains to be seen whether holidaymakers and consumers of farmed meat and nonessential imported goods will have to carry the burden of long‐term pandemic prevention to the same extent as those whose poverty or culture lead them to consume wild meat.

## 4 BALANCING THE COST OF WILD MEAT POLICY

Clearly, we continue to learn lessons from the COVID‐19 pandemic, and we must make changes to reduce the likelihood of such crises in the future. Yet, the costs of solutions should not be borne alone by those who can least afford it (Roe et al., 2020). If robust, culturally responsive cost–benefit analyses reveal that restrictions on wildlife consumption or trade are the most effective option to prevent future pandemics and reduce (rather than reallocate) loss of life, these restrictions must be place‐based, rather than unilateral. They must also be balanced by measures that avert legislated starvation, cultural extinction and exacerbated poverty (e.g., Lee et al., 2014; Lyver et al., 2019a; Milner‐Gulland & Bennett, 2003; Robinson & Bennett, 2002).

In keeping with broader Convention on Biological Diversity (2012) recommendations , we advocate for pandemic policy that:

- considers culture‐based exemptions to wild meat bans, to avoid irrevocable harm to IPLC through extensive direct and cascading impacts on multiple facets of culture and ways of life (Yletyinen et al., 2020);
- enhances local food security and availability for IPLC by alleviating other pressures on wildlife populations (e.g., reducing the reliance on bushmeat to feed urban workforce) and habitats (e.g., destruction of habitat by private resource development and extractive industries); and
- provides support from international agencies, governments, and the private sector for IPLC to develop culturally acceptable and economically feasible alternative food (and income) sources where wildlife alone cannot be sustainably used to support current or future livelihood needs. This latter recommendation could reduce subsistence use of wildlife for nutrition, though alternative foods may not address the necessity of harvests as a means of cultural transmission and knowledge production.

In conclusion, putting aside the potential ineffectiveness and difficulty of enforcing a wildlife consumption ban (Wang et al., 2020), adopting wildlife harvest or trade bans internationally could create a globally inequitable solution that disproportionately harms IPLC and people in poverty (Booth et al., 2021). In addition, the decisions surrounding wild meat policy must be made in genuine partnership with IPLC, not imposed upon them, while providing feasible pathways to protect the integrity of their culture. Solutions to complex problems are seldom simple, so we hope that both the media and policymakers will allow IPLC voices to enter the discourse on pandemic prevention, and that the world will pursue solutions that are both effective and promote equitable human rights and justice.

## AUTHOR CONTRIBUTIONS

All authors contributed to the idea development and writing of the manuscript.

## ETHICS STATEMENT

The manuscript has been submitted solely to this journal and is not published, in press, or submitted elsewhere. All the research meets the ethical guidelines, including adherence to the legal requirements of the study country.

## DATA ACCESSIBILITY STATEMENT

This manuscript presents no primary data.

## CONFLICT OF INTEREST

The authors declare no conflicts of interest.
